# Laparoscopic hysterosacropexy in case of total uterus prolapse – case report

**DOI:** 10.1016/j.ijscr.2018.10.052

**Published:** 2018-10-29

**Authors:** Paweł Szymanowski, Wioletta Katarzyna Szepieniec, Paweł Gruszecki, Isabel Netzer

**Affiliations:** Andrzej Frycz Modrzewski Krakow University, Department for Gynecology and Urogynecology, Bochenka 12, 30-693 Krakow, Poland

**Keywords:** BMI, body mass index, FDA, Food and Drug Administration, POP-Q, pelvic organ prolapse quantification system, Uterus prolapse, Apical defect, Hysteropexy, Hysterosacropexy

## Abstract

**Introduction:**

Due to the many advances in laparoscopic surgery in urogynecology, various uterus-preserving techniques are increasingly being used in treatment. The following is a report of the case of a 43-year-old female with uterine prolapse POP-Q 4. This patient successfully underwent a minimally invasive laparoscopic procedure while preserving the uterus.

**Presentation of case:**

A 43-year-old caucasian female who was suffering from prolapse of the uterus POP-Q 4, overflow incontinence, dysuria and urinary retention was admitted to our department for the purpose of diagnostic tests and operative treatment. A gynecological examination showed a protruded hernial sac with the complete, normal sized uterus which had a thick appearance. The hernial sac also contained parts of the urinary bladder and intestines. Pelvic organ prolapse was classified as follows: prolapse of the uterus POP-Q 4. After repositioning the uterus with Kristeller specula we found a cystocele POP-Q 1 and a rectocele POP-Q 1. We also discovered a defect in the perineal region. Considering the patient's age and ASA Score 1, minimally invasive treatment was proposed: laparoscopic hysterosacropexy with perineoplasty.

**Discussion:**

Transabdominal sacrocolpopexy is associated with a low recurrence rate and also lower rates of dyspareunia when compared with vaginal sacrospinal colpopexy. Both sacrocolpopexy and implantation of anterior vaginal polypropylene mesh have better success rates and a lower rate of reoperation than vaginal uterosacral suspension. In level I defects, the right approach seems to be sacropexy. In this technique, the sacrouterine ligament is restored by recreating a ligament with polypropylene mesh.

**Conclusion:**

Laparoscopic sacrohysteropexy seems to be a good alternative in the treatment of uterus prolapse. It can also be viewed as a possible alternative for apical defect POP Q III or IV.

## Highlights

• Total uterine prolapse can be treated optimally without hysterectomy.
• Use of a small mesh as a reduction of alloplastic material.
• Patient can be discharge on the next day after the operation.
• Operation time is short.

## 1 Introduction

The progress made in the advancement of laparoscopic surgery has ushered in new possibilities in urogynecology, particularly concerning the increase of techniques which can save the uterus. These techniques might be more accurately described as therapy because the reason for uterine prolapse is not a disease of the uterus, but defects or disorders in the pelvic floor, and unfortunately performing a hysterectomy still remains the most common way to treat uterine prolapse. The cause for uterine prolapse is a defect of the sacrouterine ligaments (level I defect) and this cannot be treated with a hysterectomy. Consequently, after a hysterectomy in level I defects, the recurrence rate of vaginal vault prolapse is very high. This rate of de novo prolapse after hysterectomy is high because the sacrouterine ligaments were not connected to the vaginal apex. What follows is the case of a 43-year-old female with a uterus prolapse POP-Q 4. We successfully performed a minimally invasive laparoscopic hysterosacropexy procedure with preservation of the uterus. And using this method, the uterus was able to be preserved, thus meeting patient expectations and allowing her to have a normal life.

This work has been reported in line with the SCARE criteria [9].

## 2 Presentation of case

A 43-year-old caucasian female (BMI: 27) who suffered from prolapse of the uterus POP-Q 4, overflow incontinence, dysuria, and urinary retention was admitted to our department in July 2018 in order to undergo diagnostic tests and operative treatment.

The patient’s gynecological history shows regular menstruation, three childbirths: two were vaginal deliveries (2003–2950 g; 2006–3300 g), one caesarean section (2016–3700 g); two miscarriages (in 1998 and 2000) and the removal of a left ovarian cyst via laparotomy. The first reported symptoms of prolapse occurred after the first childbirth in 2003. Previous therapy consisted of inserting a vaginal pessary. The prolapse, however, deteriorated after the second delivery in 2006. Concerning family history, the patient's mother suffered from a total prolapse of the uterus and underwent an operation at the age of 60.

During the gynecological examination, the protruded hernial sac appeared very thick and was filled with a normal-sized uterus, and parts of the bladder and intestines. The uterine cervix was found to be elongated and showed an ulceration at the top. Pelvic organ prolapse classification was quantified as follows: prolapse of the uterus POP-Q 4. After repositioning the uterus with Kristeller specula we found a cystocele POP-Q 1 and rectocele POP-Q 1. We also discovered a defect in the perineal region. Taking into consideration the patient's age and ASA Score 1, minimally invasive treatment was proposed: laparoscopic hysterosacropexy with perineoplasty. Preoperative ultrasound diagnostic revealed no pathological changes of the uterus and both adnexa, and of course no urine retention in the kidneys. No more diagnostic were used. We did not use any questionnaires about incontinence or quality of life. The overflow incontinence has been revealed by the urodynamic examination about 2 weeks before surgery.

**Fig. 1:**
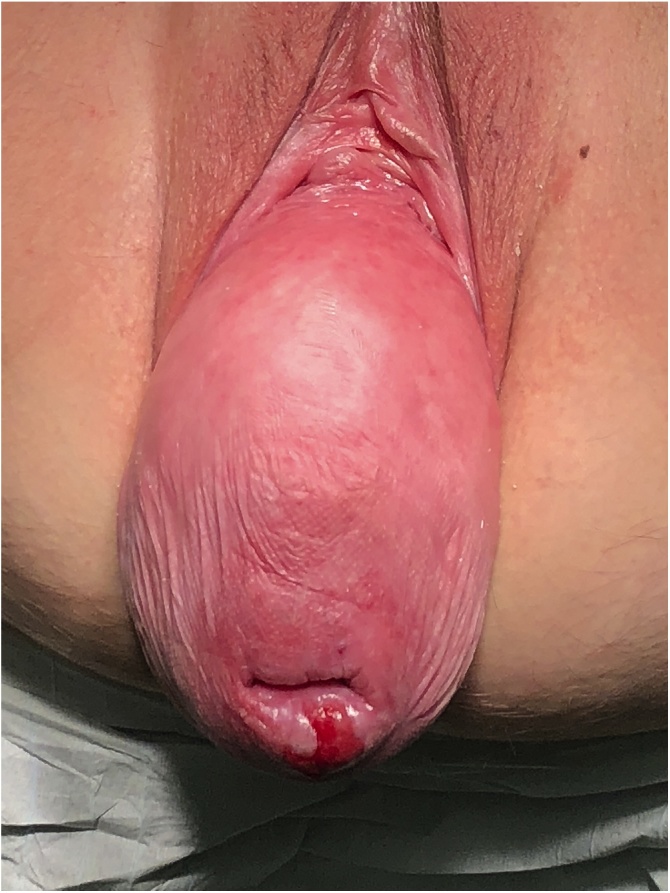
Prolaps POP-Q 4 – preoperative consideration.

**Fig. 2:**
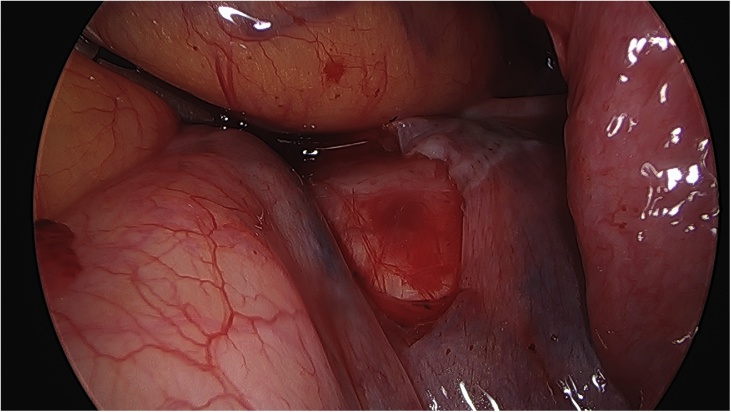
Preparation of the sacro-promontory.

**Fig. 3:**
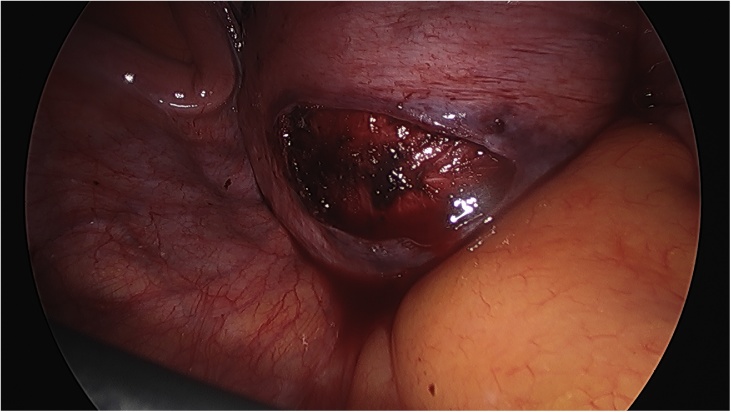
View of the posterior cervical wall after the preparation.

**Fig. 4:**
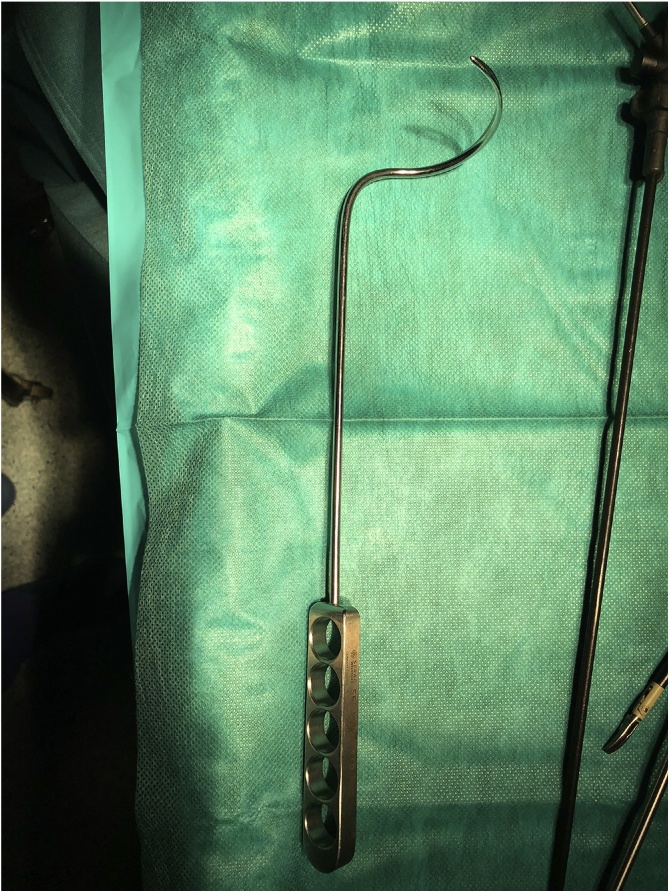
Niemeyer Helix.

**Fig. 5:**
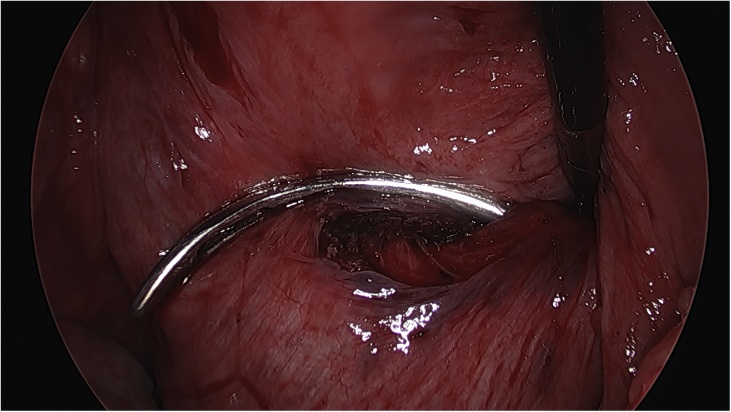
Tunnel under the peritoneum made by the Niemeyer’s helix.

**Fig. 6:**
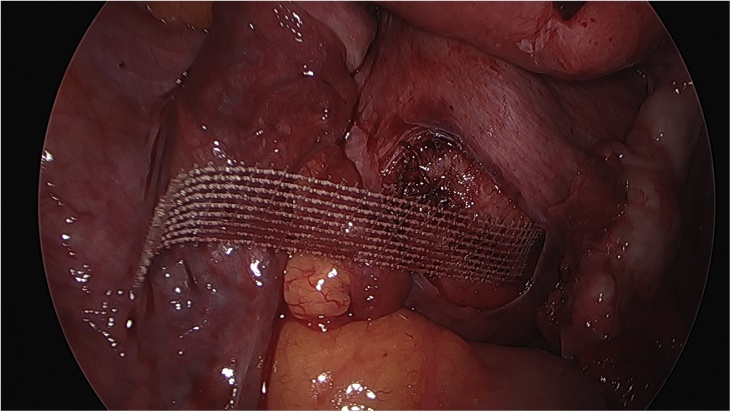
Mesh (tape) used by the operation.

**Fig. 7:**
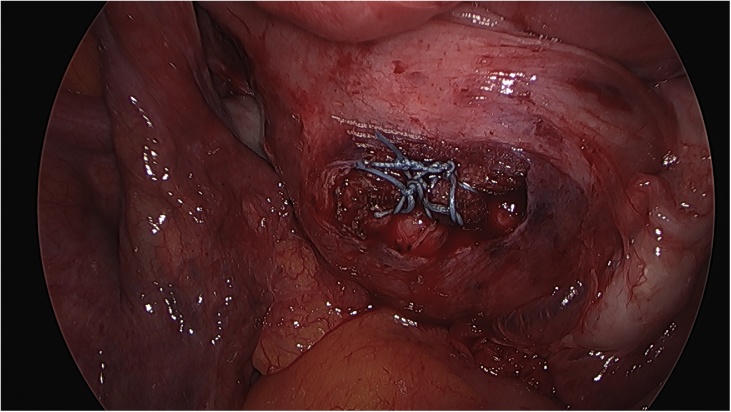
Fixation of the tape on the cervix.

**Fig. 8:**
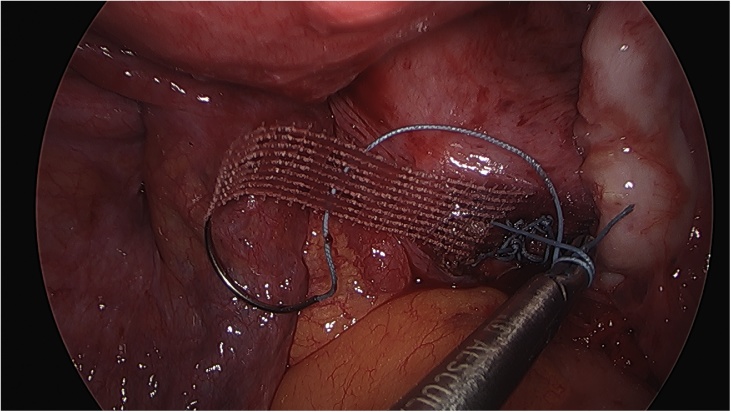
Fixation of the tape on the anterior longitudinal ligament at the sacro-promontory.

**Fig. 9:**
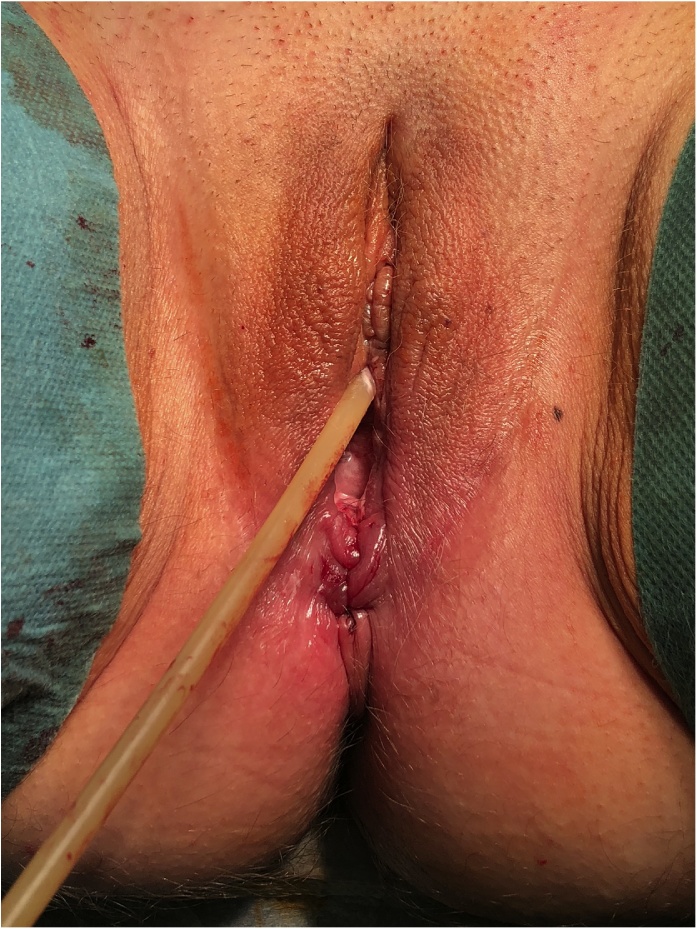
Postoperative consideration without prolapse.

On the day of the surgery, the patient was placed under general endotracheal anesthesia in a lithotomy position. After routine preparation, a sterile catheter was placed in the bladder and a bullet forceps was placed upon the cervix in order to manipulate the uterus during the procedure. A 10 mm trocar was inserted directly into an incision in the umbilical crease without the use of a Veress needle and carbon dioxide was insufflated into the peritoneum. After insertion of the 30° camera, three more trocars were placed into the lower abdomen (10 mm and 5 mm on the left side and 5 mm on the right side of the patient). A close inspection of the lower pelvic region showed unremarkable left adnexa and barely visible ones on the right that were located along with the hernial sac inside the vagina (Fig. 1). After the uterus was repositioned, there were no macroscopic changes visible. The uterus appeared to be moveable with a thick and stretched parietal peritoneum. The other organs showed no macroscopic changes. The patient was placed in a Trendelenburg position to ensure visibility of the whole pelvis. After presentation of the right ureter, the peritoneum was opened over the sacrum on the promontorial level and the anterior longitudinal ligament of the spine was exposed without injuring the inferior hypogastric plexus or the median sacral artery (Fig. 2). Next, an incision was made on the parietal peritoneum on the dorsal wall of the cervix uteri and it was partially separated from the cervix (Fig. 3). Using a Niemeyer Helix (Fig. 4), which was inserted directly through the abdominal wall, a retroperitoneal tunnel was formed between the promontorium and the cervix uteri, following the anatomical location of the right uterosacral ligament (Fig. 5). A 20 × 1 cm polypropylene mesh was used for the procedure (Fig. 6). The proximal end of the graft was pulled through the retroperitoneal tunnel. Next, the distal end of the mesh was anchored to the cervix with non-absorbable interrupted sutures 2–0 (Fig. 7). The proximal end of the graft was attached to the right side of the longitudinal anterior ligament of the sacral spine with the same type of sutures (Fig. 8). All excessive mesh was removed, so that about 4–5 cm of mesh remained. The peritoneum over the sutured mesh was carefully closed with absorbable Vicryl 2-0 sutures to prevent the intestines from contacting the graft. Hemostasis was ensured, all instruments removed from the abdomen under visual control and the incisions closed in a standard fashion. Upon placement of the bullet forceps in the area of the perineum, skin and vaginal mucosa were partially removed along the central body line. The wound was closed with continuous stitches, narrowing the vestibule of the vagina and maintaining proper anatomy (Fig. 9). The procedure was completed without complications, and complete hemostasis was ensured. On the day of discharge, the postoperative site showed no defect at level I and a cystocele POP Q I and a rectocele POP Q I at level II. A check-up two and six weeks after the operation showed the same result as at the time of discharge and good healing of the wound. Symptoms of prolapse, such as dysuria and urinary retention, had disappeared completely. Next control examinations are planned after 3, 6 and 12 months.

## 3 Discussion

It has been proven that transabdominal sacrocolpopexy is associated with lower rates of recurrence and also less dyspareunia compared to vaginal sacrospinal colpopexy. Studies have shown that sacrocolpopexy and implantation of anterior vaginal polypropylene mesh both have better outcome rates and lower reoperation rates than vaginal uterosacral suspension. In addition, the polypropylene implants are associated with postoperative complications such as erosion or pain during physical exercise and sexual intercourse. Some authors have confirmed that current evidence does not support the use of mesh repair compared with native tissue repair for anterior compartment prolapse because of the increased morbidity [1]. Due to these facts, the FDA issued a warning against vaginal mesh implantation.

Pelvic floor repair with mesh implants can not be dispensed with entirely, however. In elderly patients or in the situation of recurrence of cystocele with a lateral defect, mesh implantation is certainly an important option. If there is an indication for surgery, it is important to select the operation techniques that have the greatest chance of a full recovery.

In the case of level I defects, the appropriate surgical technique seems to be sacrocolpopexy or sacrohysteropexy. Here, the sacrouterine ligament is restored with a polypropylene mesh. This method repairs the defect and at the same time maintains cranially-directed physiological uterine mobility. A hysterectomy is not necessary because the problem lies not in the uterus itself, but in its damaged ligaments. So, a hysterectomy does not treat the defect at level I and actually complicates the patient's situation. This type of treatment very often leads to relapses in the form of vaginal vault prolapse. The incidence of vaginal vault prolapse was found to be 11.6% when a hysterectomy had been performed for genital prolapse [2]. Due to the deterioration of blood supply in the upper third of the vagina caused by a hysterectomy, the rate of mesh erosions is high and in some cases makes a subsequent sacrocolpopexy necessary. Some authors found mesh erosion rates as high as 20% after a hysterectomy and a pelvic reconstruction with a mesh [3]. The overall rate of mesh erosion after sacrocolpopexy in Nygaard et al. was 3.4% (in 70 out of 2178 cases) [4]. Other sources found the erosion rate after abdominal sacrocolpopexy to be 12% [5]. Also, in other study [6], sacral colpopexy with hysterectomy was associated with a rate of mesh erosion five times higher when compared with sacral hysteropexy.

In a systematic review of the literature, other authors found hysterosacropexy to be as effective as a vaginal hysterectomy with reduced operating time, blood loss, and recovery time [7].

The recent systematic review about the comparision between laparoscopic and abdominal sacrocolpopexy revealed no significant difference between this two groups [8]. In case of the uterine conserving surgery there is currently no papers, which compare this procedures.

For the authors of the present work, laparoscopic hysterosacropexy seems to be an effective and minimally invasive method that targets the defect directly. Larger studies are needed, however, to determine recurrence rates and complications of this procedure. The long-term results of patients with high-grade level I defects, such as POP-Q 3 and POP-Q 4, are of special interest.

## 4 Conclusion

Transvaginal surgical procedures used to correct level I defects, such as sacrospinouscolpopexy, uterosacrospinouscolpopexy or procedures using transvaginal mesh show inferior results to sacrocolpopexy. Hysterosacropexy, when performed in a minimally invasive way through laparoscopy especially in young and healthy patients who don’t have any signs of uterus disorders, should become the gold standard in the treatment of uterine prolapse and also in larger, more serious defects.

## Conflict of interest

No conflict of interest for all authors.

## Sources of funding

No payment was authorized by any source.

## Ethical approval

No specific ethical approval was necessary, because this case report is related to an operative procedure necessary to maintain the patient’s health. Specific informed consent and authorization for publication of anonymous data was obtained from the patient.

## Consent

Written informed consent was obtained from the patient for the publication of this case report and accompanying images. On request, a copy of the written consent is available for review by the Editor-in-Chief of this journal.

## Author contributions

Each author contributed to diagnosis, treatment, and postoperative follow-up of patient. Specific recording of pathologic data and an adequate review of literature was performed by each author.

## Registration of research studies

At this point, we declare that no registration is required for our case report, confirming that an internal database, with data related to the patient, is accessible with specific approval of the administrators of the Hospital.

## Guarantor

Pawel Szymanowski MD, PhD.

## Provenance and peer review

Not commissioned externally peer reviewed.

## References

[1] Maher C., Feiner B., Baessler K., Christmann-Schmid C., Haya N., Brown J. (2016). Surgery for women with anterior compartment prolapse. Cochrane Database Syst. Rev..

[2] Marchionni M., Bracco G.L., Checcucci V., Carabaneanu A., Coccia E.M., Mecacci F., Scarselli G. (1999). True incidence of vaginal vault prolapse. Thirteen years of experience. J. Reprod. Med..

[3] Carramão S., Auge A.P., Pacetta A.M., Duarte E., Ayrosa P., Lemos N.L., Aoki T. (2009). A randomized comparison of two vaginal procedures for the treatment of uterine prolapse using polypropylene mesh: hysteropexy versus hysterectomy. Rev. Col. Bras. Cir..

[4] Nygaard I.E., McCreery R., Brubaker L., Connolly A., Cundiff G., Weber A.M., Zyczynski H. (2004). Pelvic floor disorders network. Abdominal sacrocolpopexy: a comprehensive review. Obstet. Gynecol..

[5] Kohli N., Walsh P.M., Roat T.W., Karram M.M. (1998). Mesh erosion after abdominal sacrocolpopexy. Obstet. Gynecol..

[6] Gutman R., Maher C. (2013). Uterine-preserving POP surgery. Int. Urogynecol. J..

[7] Cayrac M., Warembourg S., Le Normand L., Fatton B. (2016). Does hysterectomy modifies the anatomical and functional outcomes of prolapse surgery? Clinical practice guidelines. Prog. Urol..

[8] Ichikawa M., Kaseki H., Akira S. (2018). Laparoscopic versus abdominal sacrocolpopexy for treatment of multi-compartmental pelvic organ prolapse: a systematic review. Asian J. Endosc. Surg..

[9] Agha R.A., Fowler A.J., Saetta A., Barai I., Rajmohan S., Orgill D.P., for the SCARE Group (2016). The SCARE statement: consensus-based surgical case report guidelines. Int. J. Surg..

